# Long noncoding RNA DANCR knockdown inhibits proliferation, migration and invasion of glioma by regulating miR-135a-5p/BMI1

**DOI:** 10.1186/s12935-020-1123-4

**Published:** 2020-02-18

**Authors:** Lei Feng, Tao Lin, Haijiang Che, Xiaoming Wang

**Affiliations:** Department of Neurosurgery, Xidian Group Hospital, No. 97, Fengdeng road, Lianhu District, Xi’an, 710000 Shaanxi China

**Keywords:** Glioma, DANCR, miR-135a-5p, BMI1

## Abstract

**Background:**

Glioma is the most common and aggressive primary brain tumor with high mortality rate around the world. LncRNAs have been identified to play key roles in tumorigenesis in various cancers, including glioma. However, the precise mechanism of DANCR in progression of glioma remains poorly defined.

**Methods:**

The expression levels of DANCR, miR-135a-5p and BMI1 were measured by qRT-PCR in glioma tissues and cells. Cell proliferation, migration and invasion were detected by CCK-8 assay and transwell assay, respectively. The possible binding sites of miR-135a-5p and DANCR or BMI1 were predicted by online software and verified using luciferase report assay and RNA immunoprecipitation (RIP) assay. Western blot analysis was carried out to detect the protein of BMI1 expression. A xenograft tumor model was established to investigate the functions of DANCR in glioma progression in vivo.

**Results:**

DANCR was upregulated and miR-135a-5p was downregulated in glioma tissues and cells. Knockdown of DANCR inhibited cell proliferation, migration and invasion in glioma cells. In addition, miR-135a-5p was a direct target of DANCR, and its elevated expression could reverse miR-135a-5p inhibition-mediated progression of glioma. Moreover, miR-135a-5p could specially bind to BMI1, and the expression of BMI1 was obviously elevated in glioma tissues and cells. Furthermore, DANCR acted as a ceRNA to regulate BMI1 expression and BMI1-mediated effects on progression of glioma by sponging miR-135a-5p. Besides, inhibition of DANCR limited tumor growth by regulating miR-135a-5p and BMI1 expression in vivo.

**Conclusion:**

DANCR knockdown inhibited cell proliferation, migration and invasion in glioma cells through regulating miR-135a-5p/BMI1 axis, providing viable therapeutic avenues for treatment of glioma.

## Background

Glioma is the most common and aggressive malignant tumor in the brain of adults with high mortality rate worldwide [1]. Over the last decade, despite advances in therapeutic, such as surgery, immunotherapy, stereotactic radiotherapy and new chemotherapy drugs, the prognosis of patients with glioma is remains poor [2, 3]. Thus, elucidating the molecular mechanisms of gliomas and searching effective therapeutic strategies are of great significance for the patients with glioma.

Long non-coding RNAs (lncRNAs) are a class of non-coding RNAs that are more than 200 nucleotides in length without significant protein-coding capacity [4]. LncRNAs have been identified to play key roles in tumorigenesis in various cancers, including glioma [5]. For example, lncRNA CRNDE has been proved as a diagnostic marker for glioma or a potential therapeutic target for the treatment of glioma [6]. Long non-coding RNA ENST00462717 has been shown to inhibit proliferation, survival and migration in glioma via suppressing the MDM2/MAPK signaling pathway [7]. Moreover, extensive reports have demonstrated that lncRNA Differentiation Antagonizing Non-Protein Coding RNA (DANCR) is a newly identified oncogenic lncRNA, and it is reported to promote the invasion of prostate cancer, colorectal cancer and gastric cancer, etc. [8–10]. In addition, it has been proved that high expression of DANCR predicts poor prognosis for glioma patients [11]. However, the precise mechanism of DANCR in progression of glioma remains poorly defined.

Increasing evidence has suggested that lncRNA can be as a competing endogenous RNA (ceRNA) to regulate the expression of targeted genes by sponging microRNA [12]. In recent years, a plenty of reports have strongly suggested that aberrant expression of microRNA (miRNA) is a feature of human glioblastoma [13, 14]. However, lncRNA and miRNA network in glioma is still largely unknown and needs further investigation. MiR-135a-5p, a miR-135 family member, has been confirmed as a tumor suppressor in many cancers, including glioma [15]. However, there is no evidence in support of the interaction between DANCR and miR-135a-5p in glioma. B-lymphoma Moloney murine leukemia virus insertion region-1 (BMI1), a self-renewal gene, has been suggested to be overexpressed in various human cancers, such as ovarian cancer, colorectal cancer, lung cancer, and breast cancer [16–19]. In addition, a previous study indicated that BMI1 was a potential oncogene associate with tumorigenesis and cancer progression in glioma [20]. But the potential role and mechanism of BMI1 related to the development of glioma remain unclear.

In this study, we explored the expression level of DANCR in glioma tissues and cells. Moreover, we investigated the ceRNA regulatory network of DANCR/miR-135a-5p/BMI1, and explored their possible effects on cell proliferation, migration and invasion, providing a novel diagnostic and strategy for treatment of glioma.

## Materials and methods

### Patients specimens

In this study, a total of 33 glioma tissues and normal tissues were kindly provided by Xidian Group Hospital. All subjects had signed informed consent and this study was granted by the Ethics Committee of Xidian Group Hospital. All tissue samples were collected immediately after resection and frozen in liquid nitrogen, and then preserved at − 80 °C until experiments were carried out.

### Cell culture and transfection

Human glioma cell line LN229 was purchased from American Tissue Culture Collection (ATCC, Manassas, VA, USA). Human glioma cell line U251 and normal human astrocytes (NHAs) were bought from the Chinese Academy of Sciences (Shanghai, China). The cells were cultured in RPMI-1640 medium (Invitrogen, Carlsbad, CA, USA) supplemented with 10% fetal bovine serum (FBS; Gibco, Carlsbad, CA, USA) at 37 °C under a humidified atmosphere containing 5% CO_2_.

Small interfering RNA (siRNA) against DANCR (si-DANCR), siRNA negative control (sh-NC), miR-135a-5p mimic (miR-135a-5p), miR-negative control (miR-NC), miR-135a-5p inhibitor (in-miR-135a-5p), and in-miR-NC were purchased from GenePharma Co., ltd (Shanghai, China). The pcDNA-DANCR (DANCR) overexpression plasmid and pcDNA-BMI1 (BMI1) overexpression plasmid were bought from Hanbio Biotechnology Co., ltd (Shanghai, China). The sequences were list as follows: si-DANCR#1: 5′-GCGTACTAACTTGTAGCAA-3′, si-DANCR#2: 5′-CTACAGGCACAAGCCATTG-3′, si-DANCR#3: 5′-GCUGGUAUUUCAAUUGACU-3′, si-NC: 5′-UUCUCCGAACGUGUCACGUTT-3′, miR-135a-5p mimic: 5′-UAUGGCUUU UUAUUCCUAUGUGA-3′, miR-NC: 5′-UCUCCAAACGUGUCACCUTT-3′, miR-135a-5p inhibitor: 5′-UCACAUAGGAAUAAA AAGCCAUA-3′, in-miR-NC: 5′-CAGUACUUUUGUGUAGUACAA-3′. A pair of specific primers (forward 5′-CAAGGATCCGCCCTTGCCCAGAGTCTTCC-3′ and reverse 5′-CCGCTCGAGGTCAGGCCAAGTAAGTTTAT-3′) was used to amplify DANCR. Then the amplified products were inserted into pcDNA3.1 plasmids between BamHI and XhoI restriction enzyme sites to induce the overexpression of DANCR. The coding sequences of BMI1 (forward, 5′-CTAGCTAGCATGCATCGAACAACGAGAATCA-3′ and reverse 5′-CCGCTCGAGTCAACCAGAAGAAGTTGCTGATG-3′) was amplified and then cloned into PCDNA3.1 vector. LN229 and U251 cells were transfected with oligonucleotides or vectors using Lipofectamine 3000 (Invitrogen) following the manufacturer’s protocol.

### qRT-PCR

Total RNA was isolated from tissue samples or cells using a Trizol reagent (Thermo Fisher Scientific, Waltham, MA, USA). The first-strand cDNA for miR-135a-5p, BMI1 and DANCR was synthesized from total RNA using MicroRNA Reverse Transcription Kit and TaqMan Reverse Transcription Kits (Applied Biosystems, Foster City, CA, USA), respectively. The first-strand cDNA was used to carry out qRT-PCR with 2× SYBR Green qPCR Mix (Aidlab, Beijing, China) on an ABI 7900HT sequence detection machine (Thermo Fisher Scientific). The primers used were presented as below: for DANCR, 5′-GCCACAGGAGCTAGAGCAGT-3′ (forward) and 5′-GCAGAGTATTCAGGGTAAGGGT-3′ (reverse); for miR-135a-5p, 5′-CCGGCGTATGGCTTTTTATTCC-3′ (forward) and 5′-CAGTGCAGGGTCCGAGGT-3′ (reverse); for BMI1 5′-ACTGGAAAGTGACTCTGGGA-3′ (forward) and 5′-TACTGGGGCTAGGCAAACAA-3′ (reverse); for GAPDH, 5′-TATGATGATATCAAGAGGGTAGT-3′ (forward) and 5′-TGTATCCAAACTCATTGTCATAC-3′ (reverse); for U6 (Forward, 5′-CTCGCTTCGGCAGCACATATACT-3′ (forward) and 5′-ACGCTTCACGAATTTGCGTGTC-3′ (reverse). GAPDH was deemed as a reference gene of BMI1 and DANCR, while U6 was an internal control of miR-135a-5p. The relative expression levels of miR-135a-5p, BMI1 and DANCR were evaluated with 2^−ΔΔCt^ method.

### CCK-8 assay

Cell proliferation was analyzed using the Cell Counting Kit-8 (Beyotime, Jiangsu, China). In brief, LN229 and U251 cells (5 × 10^3^ cells/well) were incubated in 96 well plates for 12 h. Each well was added to CCK-8 solution at 24, 48 or 72 h after the transfection. Then, the plates were incubated for 1 h at 37 °C. Cells proliferation were detected at 450 nm using a microplate reader (Molecular Devices, Sunnyvale, CA, USA).

### Transwell assay

Migrated and invasive abilities of cells were detected using 24-well transwell chambers with 8-μm pore size polycarbonate membranes (BD Biosciences, Franklin Lakes, NJ). For cell invasion, the transwell chambers were pre-coated with Matrigel (BD Biosciences). LN229 and U251 cells in 100 μL of serum-free RPMI-1640 medium were seeded into the upper chambers (Costar, Corning, NY, USA), and 500 μL of RPMI-1640 medium supplemented with 10% FBS was added into the lower chambers. After incubation for 24 h at 37 °C with 5% CO_2_, the non-invaded cells were carefully removed from the upper chamber with a cotton swab. Next, invasive cells on lower surfaces were fixed with paraformaldehyde (Sigma, St. Louis, MO, USA) and stained with 0.5% crystal violet (Sigma) in darkness for 20 min. For cell migration the experiment was performed following the similar approach except that the membranes of transwell chambers without Matrigel.

### Dual-luciferase reporter assay

The putative binding sites of miR-135a-5p and DANCR or BMI1 were predicted by StarBase v2.0 or Tarbase v8.0 software online. Partial fragment of wild type DANCR (DANCR-WT), mutant DANCR (DANCR-MUT), 3′ untranslated regions of BMI1 wild type (BMI1 3′UTR-WT) or 3′ untranslated regions of BMI1 mutant (BMI1 3′UTR-MUT) containing the putative binding sites of miR-135a-5p were amplified and cloned into pmirGLO vectors (Promega, Madison, WI, USA). The miR-NC or miR-135a-5p was co-transfected with luciferase reporter plasmids into LN229 and U251 cells according to the manufacturer’s protocols. After transfection for 48 h, luciferase activity was examined using the Dual-Luciferase Assay System (Promega), and normalized to Renilla luciferase activity.

### RNA immunoprecipitation (RIP) assay

The relationship between miR-135a-5p and DANCR or BMI1 was performed using a Magna RNA-binding protein immunoprecipitation kit (Millipore). Briefly, LN229 and U251 cells transfected with miR-135a-5p or miR-NC for 48 h. After that, the cell lysate was incubated with a RIP buffer containing magnetic beads bounded with human anti-Ago2 antibody or IgG antibodies. Finally, the RNA in beads complexes was isolated and analyzed by qRT-PCR to demonstrate the presence of the binding targets.

### Western blot

Cells or tissues were collected and lysed in RIPA lysis buffer (Thermo Fisher Scientific) containing protease inhibitor (Sigma), followed by centrifugation at 12,000 rpm for 15 min to collect the supernatant of the total protein. Then the protein was quantified though bicinchoninic acid (BCA) protein assay kit (Beyotime). Next, equal amounts of the protein lysates were loaded onto the 10% SDS-PAGE gels, and then the protein gels were transferred to 0.22 μm polyvinylidene difluoride (PVDF) membranes (Millipore, Billerica, MA, USA). After blocking in TBST containing 5% nonfat dry milk, the membranes were incubated with primary antibodies against BMI1 (1:1000; #6964) and β-actin (1:1000; #4970) (Cell Signaling Technology, Beverly, MA, USA) overnight at 4 °C. Subsequently, the membranes were incubated HRP-conjugated secondary antibodies (Sangon Biotech, Shanghai, China) at 1:4000 dilutions for 2 h at room temperature). Finally, protein bands were visualized using ECL (Amersham Biosciences, Freiburg, Germany). The ImageJ software was used to quantify the densitometry of the bands.

### Tumor xenograft model

Lentivirus-mediated shRNA interference targeting DANCR (sh-DANCR; 5′-AGCCAACTATCCCTTCACTTACA-3′) and sh-NC (5′-TTCTCCGAACGTGTCACGT-3′) were constructed by Genechem (Shanghai, China) and then transfected into LN229 cells. Stably transfected cells (1 × 10^6^) were inoculated subcutaneously in BALB/c nude mice (n = 7/group, male, 6-week-old, Henan Experimental Animals Centre, Zhengzhou, China). The animal experiments were approved by the Animal Care and Use Committee of Xidian Group Hospital and were carried out accordance with the institutional guide for the care and use of laboratory animals. Tumor volumes were examined every 7 days with a caliper for four times and calculated following the equation: volume = (length × width^2^)/2. All mice were sacrificed after injection for 28 days, tumor specimens were weighted and collected for further analysis.

### Statistical analysis

All statistical analyses were performed with the GraphPad Prism Software (GraphPad. Software, Inc., USA). All experiments were repeated at least three times and presented as the mean ± standard deviation (SD). Kaplan–Meier method was used to plot survival curve. Correlations were detected by Spearman rank correlation. Differences between the expression levels of ANRIL, miR-135a-5p BMI1 in tumor and non-tumor tissues were compared with the Wilcoxon signed-rank test. Student’ s *t*-test were measured the differences between two groups. The differences were considered to be statistically significantly at *P *< 0.05.

## Results

### DANCR was upregulated in glioma tissues and cells, and indicated poor prognosis for glioma patients

**Fig. 1:**
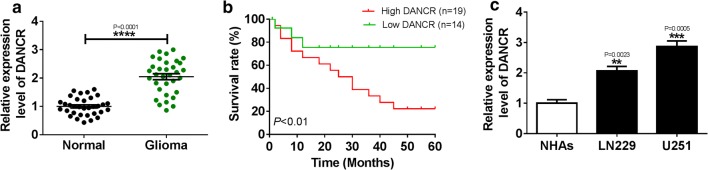
DANCR was enhanced in glioma tissues and cells, and indicated poor prognosis for glioma patients. **a** The level of DANCR was detected in glioma tissues (n = 33) and normal tissues (n = 33) by qRT-PCR. Statistical difference was analyzed by Wilcoxon signed–rank test. **b** The association of DANCR expression and prognosis for glioma patients. **c** The abundance of DANCR was analyzed in glioma cells and NHAs cells by qRT-PCR.**P < 0.01, ***P < 0.001, ****P < 0.0001

To investigate the potential roles of DANCR in glioma, qRT-PCR was perform in 33 pairs of glioma tissues and cells. We found that the level of DANCR expression was drastically higher in glioma tissues than normal tissues (Fig. 1a). Moreover, the correlation between DANCR expression and prognosis of glioma patients was measured, and patients with high DANCR expression (n = 19) had poor survival compared with low DANCR expression (n = 14) (Fig. 1b). Similarly, DANCR expression was also upregulated in LN229 and U251 cells related to NHAs cells (Fig. 1c). Furthermore, the correlation between the relative expression of DANCR and clinicopathological parameters in patients with glioma was analyzed. As shown in Additional file 1: Table S1, high expression level of DANCR was positively associated with clinical grading and tumor size, but not with age, sex, isocitrate dehydrogenase (IDH), and 6-methyl-guanine methyl transferase (MGMT). All these results suggested that high expression of DANCR was associated with glioma progression, and promoted us to explore its biological function more deeply.

### Knockdown of DANCR inhibited cell proliferation, migration and invasion in glioma cells

**Fig. 2:**
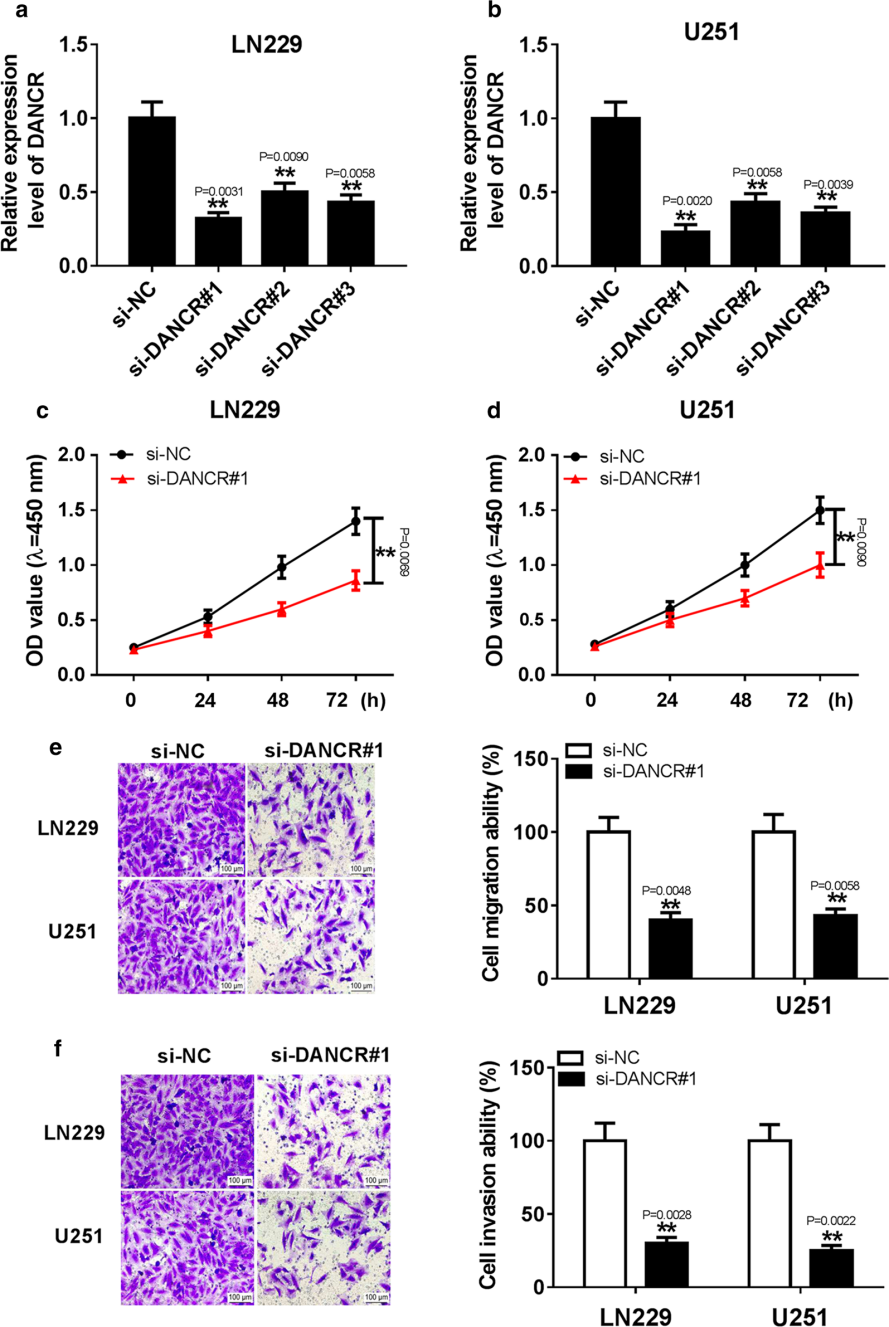
Knockdown DANCR suppressed cell proliferation, migration and invasion in glioma cells. **a**, **b** The expression of DANCR was measured in LN229 and U251 cells transfected with three independent DANCR specific si-RNAs or si-NC by qRT-PCR. **c**–**f** The effects of DANCR inhibition on cell proliferation, migration and invasion were explored in LN229 and U251 cells transfected with si-DANCR#1 or si-NC by CCK-8 assay or transwell assay respectively. **P < 0.01

To explore the effect of DANCR on glioma progression, the LN229 and U251 cells were transfected with three independent DANCR specific siRNAs. As displayed in (Fig. 2a, b), the abundance of DANCR was greatly decreased in the LN229 and U251 cells transfected with DANCR specific siRNAs. In addition, DANCR expression was the lowest in the si-DANCR#1-transfected cells. Thus, si-DANCR#1 was used in subsequent experiments. In addition, CCK-8 assay indicated that DANCR interference significantly inhibited cell proliferation at different time points in LN229 and U251 cells (Fig. 2c, d). Moreover, transwell assay was used to determine the migration and invasion abilities of glioma cells after transfection of si-DANCR for 24 h. As shown in (Fig. 2e, f), DANCR downregulation prominently inhibited the cell migration and invasion of LN229 and U251 cells. Altogether, these data indicated that DANCR played an essential role in glioma cell proliferation, migration and invasion.

### MiR-135a-5p was a direct target of DANCR

**Fig. 3:**
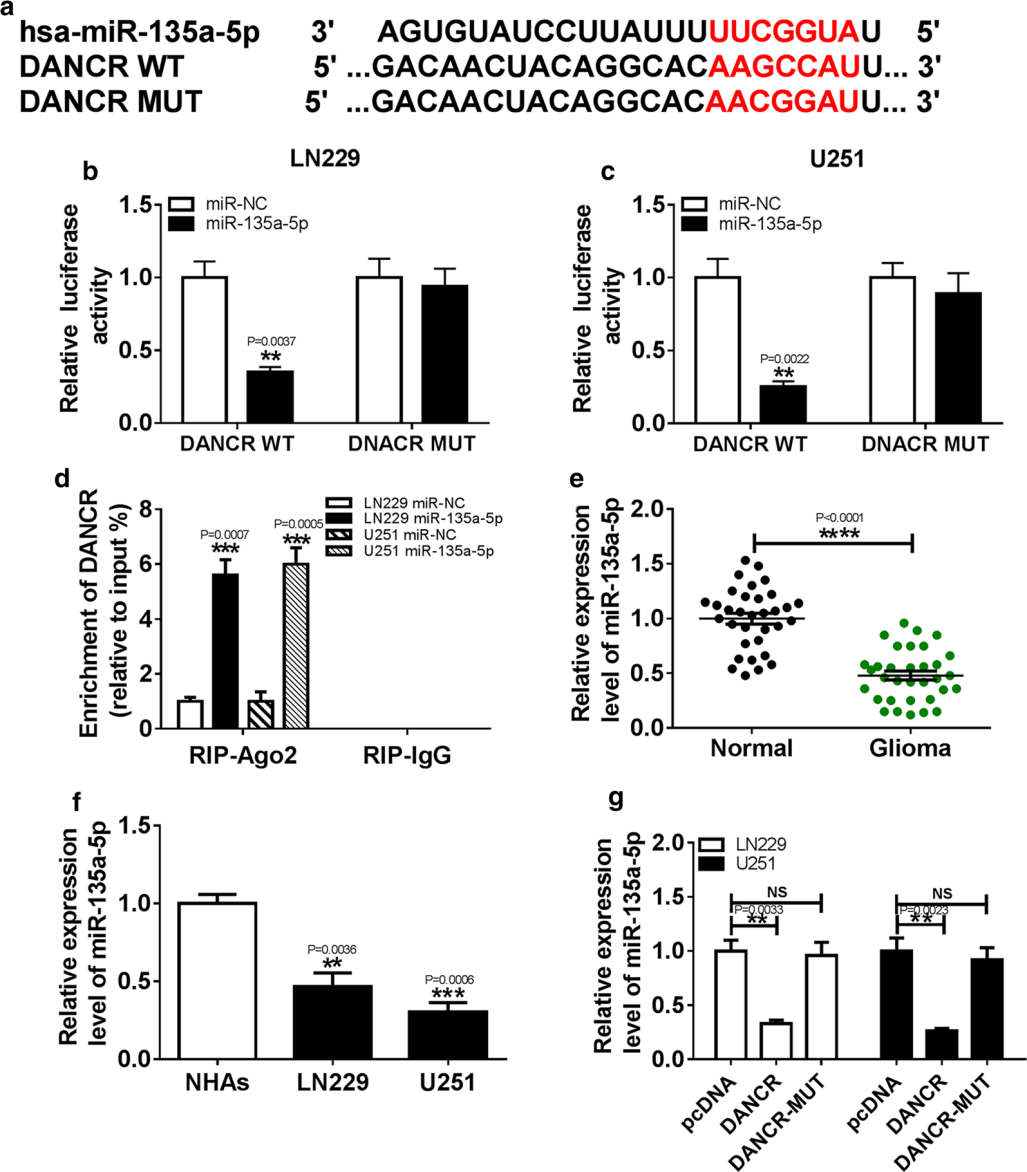
MiR-135a-5p was a direct target of DANCR. **a** The potential binding sites of DANCR and miR-135a-5p were predicted by starBase v2.0. **b**, **c** The luciferase activity was measured in LN229 and U251 cells co-transfected with DANCR-WT or DANCR-MUT and miR-135a-5p or miR-NC. **d** RIP assay was performed to detect DANCR enrichment level in IgG or Ago2 immunoprecipitation complex of LN229 and U251 cells. **e** The level of miR-135a-5p was examined in glioma tissues (n = 33) and normal tissues (n = 33) by qRT-PCR. Statistical analyses were conducted by Wilcoxon signed–rank test. **f** The abundance of miR-135a-5p was analyzed in glioma cells and NHAs cells by qRT-PCR. **g** The expression of miR-135a-5p was analyzed in LN229 and U251 cells transfected with DANCR, pcDNA, or DANCR-MUT. **P < 0.01, ***P < 0.001, ****P < 0.0001

Many lncRNAs have been reported may function as ceRNAs to regulate miRNAs and hence functionally liberates other RNA transcripts. Web-based tool Starbase v2.0 was used to predict the potential miRNA binding sites of DANCR. We found that miR-135a-5p was formed complementary bases pairing with DANCR (Fig. 3a). Subsequently, the prediction was confirmed by luciferase activity and RIP analysis. From the results we can see, overexpression of miR-135a-5p greatly decreased luciferase activity in LN229 and U251 cells transfected with DANCR-WT, whereas no obvious changes were observed in luciferase activity of DANCR-MUT reporter (Fig. 3b, c). Moreover, RIP assay showed that the LN229 and U251 cells transfected with miR-135a-5p obviously enhanced the enrichment of DANCR in Ago2 group compared with that in IgG group (Fig. 3d). Furthermore, the expression level of miR-135a-5p was downregulated in glioma tissues with respect to normal tissues (Fig. 3e). In addition, the association between the relative expression of miR-135a-5p and clinicopathological parameters in patients with glioma was assessed. As displayed in Additional file 1: Table S2, low expression level of miR-135a-5p was significantly associated with clinical grading and tumor size, but not with age, sex, IDH, and MGMT. Similarly, compared with NHAs cells, the expression of miR-135a-5p was evidently reduced in LN229 and U251 cells (Fig. 3f). In addition, overexpression of DANCR conspicuously decreased the level of miR-135a-5p expression, while transfection of DANCR-MUT has no significant effect on miR-135a-5p expression in LN229 and U251 cells (Fig. 3g). These results illustrated that DANCR directly interacted with miR-135a-5p and was negatively correlated with miR-135a-5p.

### Overexpression of DANCR reversed miR-135a-5p inhibition-mediated progression of glioma

**Fig. 4:**
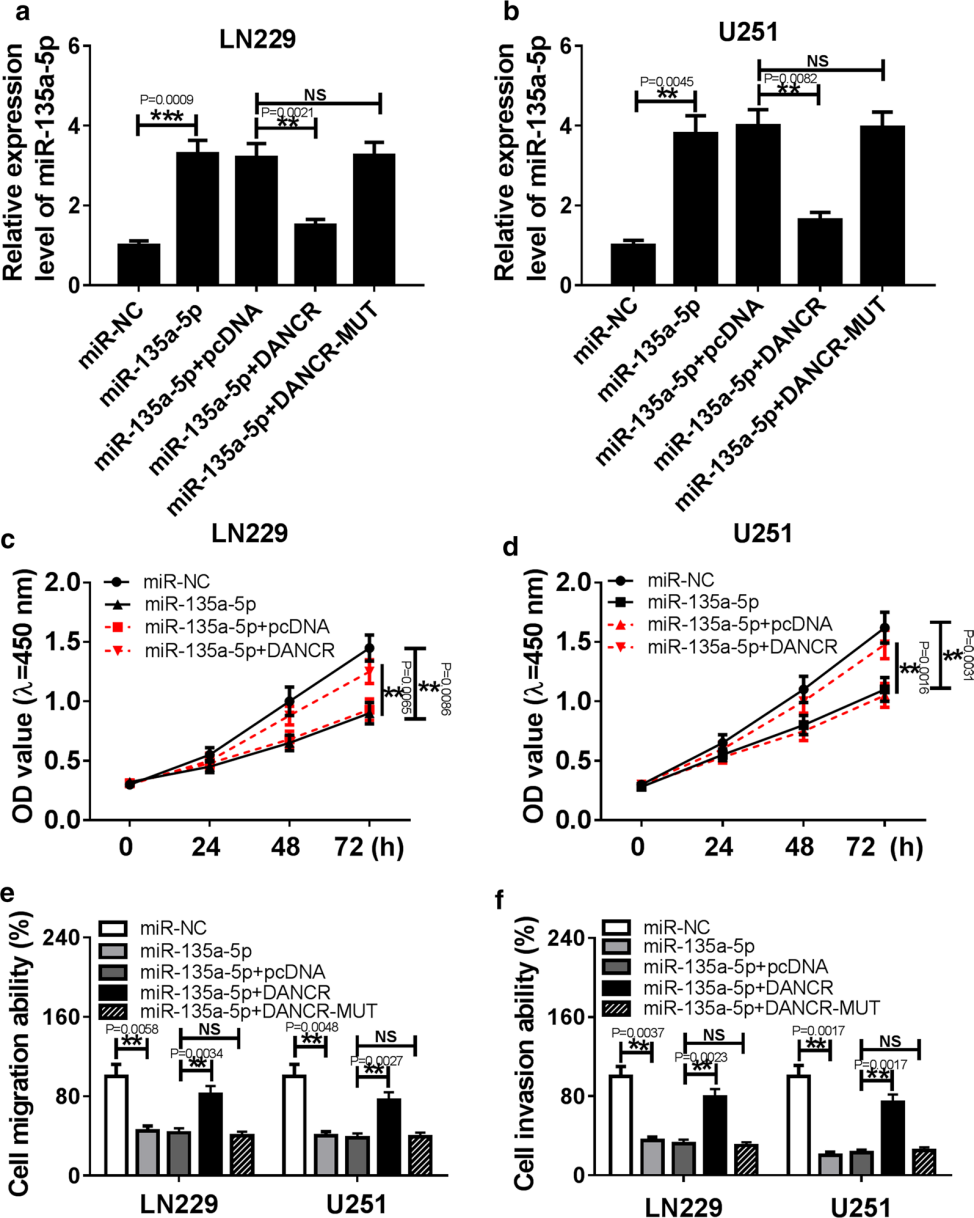
Enforced expression of DANCR abolished inhibitory effect of miR-135a-5p overexpression on progression of glioma. **a**, **b** qRT-PCR was used to detect the miR-135a-5p expression in LN229 and U251 cells transfected with miR-NC, miR-135a-5p, miR-135a-5p + pcDNA, miR-135a-5p + DANCR, or miR-135a-5p + DANCR-MUT. **c**, **d** CCK-8 assay was performed to determine the proliferation of LN229 and U251 cells transfected miR-NC, miR-135a-5p, miR-135a-5p + pcDNA, or miR-135a-5p + DANCR. **e**, **f** Cell migration and invasion were analyzed in LN229 and U251 cells transfected miR-NC, miR-135a-5p, miR-135a-5p + pcDNA, miR-135a-5p + DANCR, or miR-135a-5p + DANCR-MUT by transwell assay. **P < 0.01, ***P < 0.001

To further explore the relationship between miR-135a-5p and DANCR in glioma, miR-NC, miR-135a-5p, miR-135a-5p+pcDNA, or miR-135a-5p+ DANCR was transfected into LN229 and U251 cells. The results showed that restoration of miR-135a-5p resulted in a significant increase of miR-135a-5p expression in LN229 and U251 cells, which was abrogated by addition of DANCR but not DANCR-MUT (Fig. 4a, b). Moreover, enforced expression of miR-135a-5p obvious inhibited cell proliferation, migration and invasion in LN229 and U251 cells, whereas overexpression of DANCR abolished these effects (Fig. 4c–f). However, cell migration and invasion abilities were not evidently affected after transfection with DANCR-MUT in LN229 and U251 cells (Fig. 4e, f). These data indicated that miR-135a-5p might act as a putative tumor suppressor in glioma, and enforced expression of DANCR alleviated the inhibitory effect of miR-135a-5p overexpression on progression of glioma.

### BMI1 was a downstream of miR-135a-5p in glioma cells

**Fig. 5:**
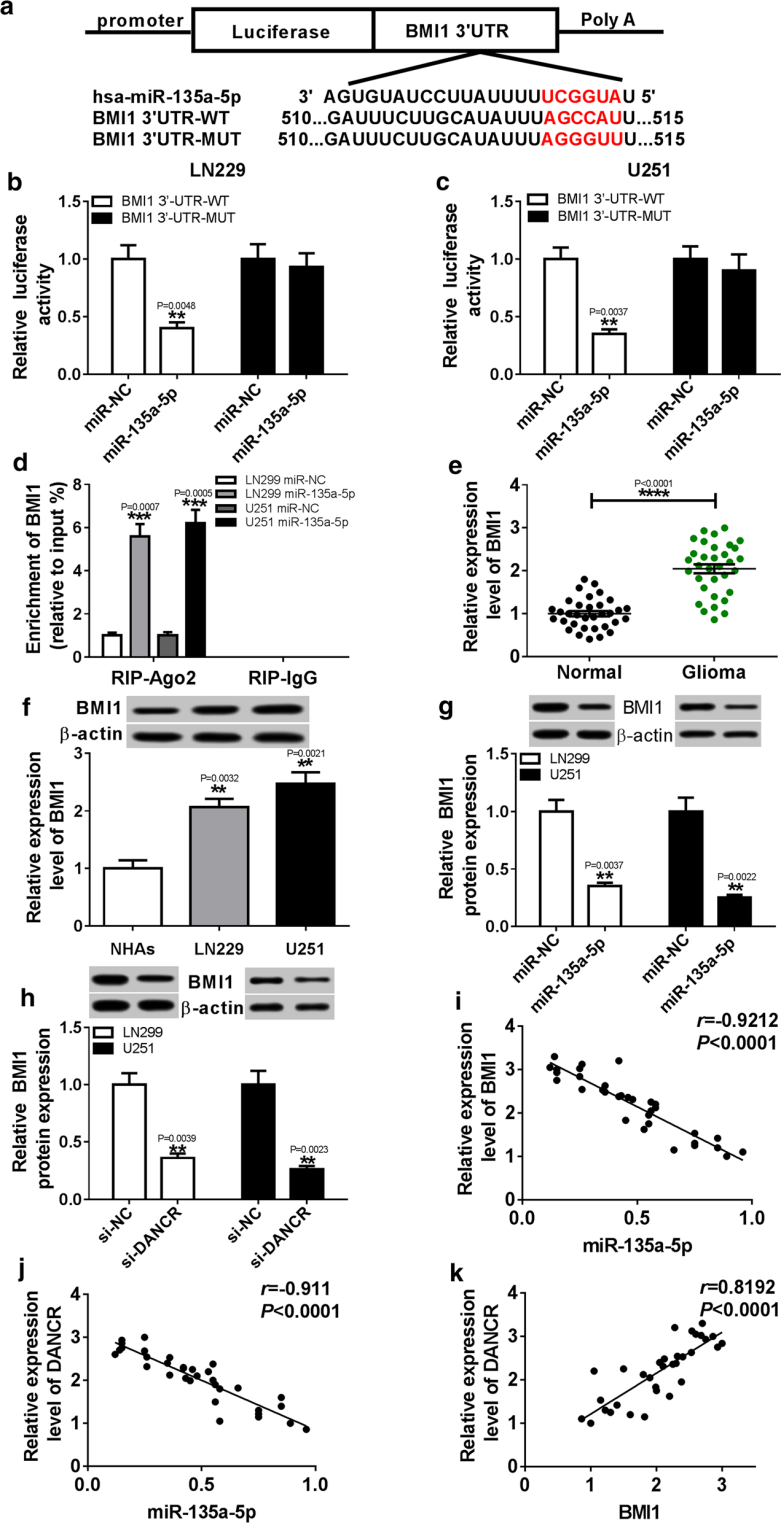
DANCR regulated BMI1 expression by sponging miR-135a-5p in glioma cells. **a** The putative binding sites of BMI1 and miR-135a-5p were predicted by Tarbase v8.0. **b**, **c** Luciferase activity was measured in LN229 and U251 cells co-transfected with BMI1 3′UTR-MUT or BMI1 3′UTR-WT and miR-135a-5p or miR-NC. **d** The enrichment of BMI1was detected in LN229 and U251 cells transfected with miR-135a-5p or miR-NC after RIP assay. **e** The abundance of BMI1was measured in glioma tissues and normal tissues by qRT-PCR. The statistical significance of differences was determined using the Wilcoxon signed-rank test. **f** The abundance of BMI1 protein was analyzed in glioma cells and NHAs cells by western blot. **g**, **h** The protein of BMI1 expression was measured in LN229 and U251 transfected with si-DANCR or si-NC by western blot. **i** The association between BMI1 mRNA level and miR-135a-5p abundance was analyzed in glioma tissues. **j**, **k** The correlation between DANCR mRNA level and miR-135a-5p or BMI1 expression was measured in glioma tissues. **P < 0.01, ***P < 0.001, ****P < 0.0001

As miRNAs primarily exert their functions by regulating expression of their downstream target genes. Tarbase v8.0 was used to search for the potential regulatory targets of miR-135a-5p, we found that BMI1 was a potential binding target of miR-135a-5p (Fig. 5a). Dual-luciferase reporter and RIP assays were performed to assess the effect of miR–135a-5p on BMI1 activity. Results showed that LN229 and U251 cells transfected with miR-135a-5p had no great effect on luciferase activity in the BMI1 3′UTR-MUT plasmids, but produced an obvious reduction in luciferase activity in the BMI1 3′UTR-WT plasmids compared with the miR-NC group (Fig. 5b, c). Besides, transfection of miR-135a-5p led to led to an evident increase of BMI1 enrichment in Ago2 group compared with that in IgG group (Fig. 5d). In addition, the expression level of BMI1 was markedly upregulated in glioma tissues in contrast to normal tissues, and the level of BMI1 protein was significantly elevated in LN229 and U251 cells relative to NHAs cells (Fig. 5e, f). Moreover, LN229 and U251 cells transfected with miR-135a-5p or si-DANCR led to an obvious reduction of BMI1 protein expression compare with control group (Fig. 5g, h). Furthermore, we also analyzed the relationship among miR-135a-5p, DANCR and BMI1, and found that expression levels of BMI1 and DANCR were negatively correlated with miR-135a-5p abundance in glioma tissues, and expression of DANCR was positively correlated with BMI1 level (Fig. 5i–k). These findings suggested that miR-135a-5p directly targeted BMI1 in glioma cells.

### DANCR regulated BMI1-mediated effects on progression of glioma via sponging miR-135a-5p

**Fig. 6:**
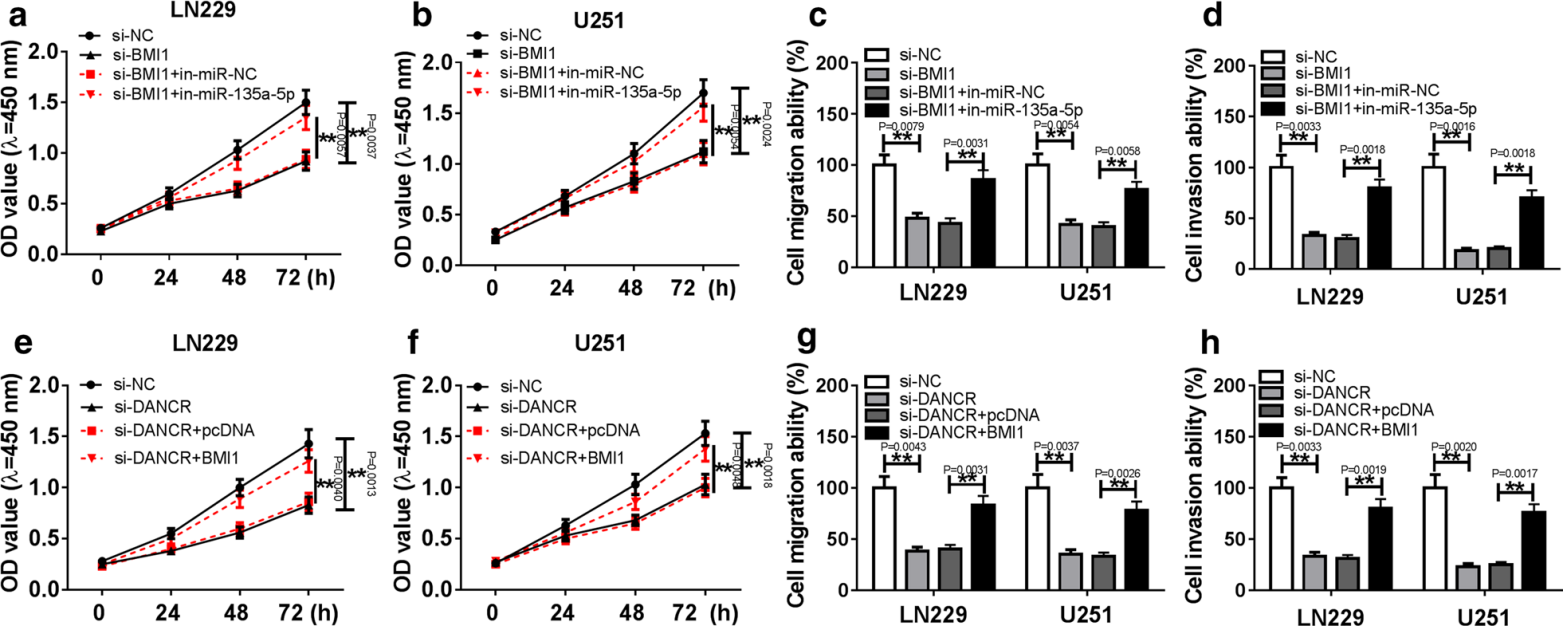
DANCR regulated effects of BMI1 on progression of glioma cells by sponging miR-135a-5p. **a**–**d** The effects of BMI1 and miR-135a-5p on cell proliferation, migration and invasion were explored in LN229 and U251 cells transfected with si-BMI1, si-NC, anti-miR-135a-5p or anti-miR-NC by CCK-8 assay or transwell assay respectively. **e**–**h** The effects of BMI1 and DANCR on cell proliferation, migration and invasion were investigated in LN229 and U251 cells transfected with si-DANCR, si-NC, BMI1 or pcDNA by CCK-8 assay or transwell assay respectively. **P < 0.01

To further investigate the effect of DANCR and miR-135a-5p on BMI1 functions in glioma cells, CCK-8 and transwell analysis were used to detect the cell proliferation and invasion in LN229 and U251 cells, respectively. As revealed in Fig. 6(a–d), knockdown of BMI1 apparently inhibited cell proliferation, migration and invasion in LN229 and U251 cells, whereas it was ablated by inhibition of miR-135a-5p. In addition, abrogation of DANCR notably suppressed cell proliferation, migration and invasion in LN229 and U251 cells, which would be abolished by overexpression BMI1 (Fig. 6e–h). These data proved that DANCR could regulate BMI1 functions by sponging miR-135a-5p.

### Knockdown of DNACR inhibited tumor growth by regulating miR-135a-5p and BMI1

**Fig. 7:**
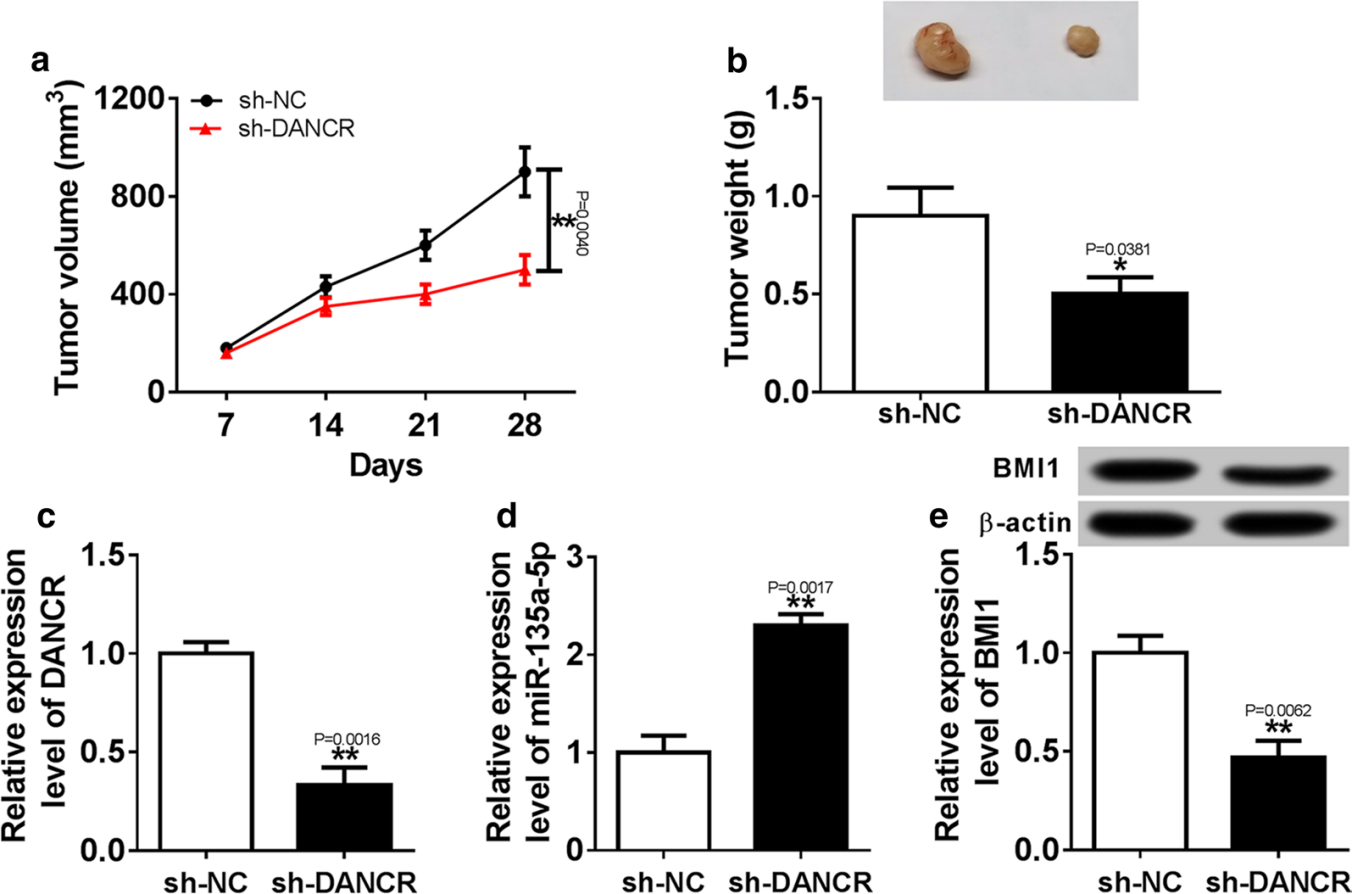
Deficiency of DANCR suppressed tumor growth by regulating miR-135a-5p and BMI1 expression in xenograft model. **a**, **b** The LN229 cells introduced with sh-DANCR or sh-NC were injected subcutaneously into nude mice, tumor volume and weight were measured in xenograft model. **c**, **d** The expressions of DANCR and miR-135a-5p were measured in xenograft tumors by qRT-PCR. **e** The abundance of BMI1 protein was detected in xenograft tumors by western blot.*P < 0.05, **P < 0.01

To explore the effect of DNACR on glioma in vivo, LN229 cells transfected with sh-DNACR or sh-NC were subcutaneously introduced into nude mice. As seen in (Fig. 7a, b), knockdown of DNACR inhibited tumor volume and weight in LN229 xenograft model. Moreover, qRT-PCR analysis showed the abundance of DNACR dramatically decreased in sh-DNACR group compared with sh-NC group (Fig. 7c). In addition, the expression of miR-135a-5p was evidently higher in sh-DNACR group than sh-NC group (Fig. 7d). Furthermore, deficiency of DNACR obviously decreased the protein level of BMI1 in xenograft tumor tissues (Fig. 7e). Thus, the findings indicated that DNACR knockdown inhibited tumor growth of glioma in vivo.

## Discussion

Due to the high migration and invasion abilities, the recurrence rate and mortality rate of glioma patients are still high and the median survival is less than 14 months [21, 22]. Recently, many lncRNAs were ectopic in glioma and might be used to identify effective therapeutic targets for glioma patients [23]. Therefore, it is of great significance to understand more about the underlying molecular mechanisms of lncRNAs for the treatment of glioma.

Recently, DANCR has been shown to be frequently dysregulated in different cancers and have plenty functions in a wide range of biological processes, including cell proliferation, cell apoptosis, cell autophagy, cell migration, invasion, and differentiation [24, 25]. In addition, DANCR was upregulated in human prostate cancer tissues and cell lines and promoted invasion of prostate cancer by repressing expression of TIMP2/3 [8]. Moreover, DANCR was indicated to contribute to migration and invasion in gastric cancer cells via inhibition of lncRNA-LET [10]. In our study, we found that the abundance of DANCR was also increased in glioma tissues and cells, and patients with high DANCR expression had poor survival compared with low DANCR expression, which is consistent with previous reports [11]. Moreover, inhibition of DANCR significantly suppressed cell proliferation, migration and invasion in LN229 and U251 cells. Furthermore, DNACR knockdown inhibited tumor growth of glioma in vivo. These results proved that DNACR might act as a diagnostic biomarker in glioma.

Mounting evidence suggested that lncRNAs could function as miRNA sponges or decoys to regulate the expressions of miRNAs targets genes in different types of cancer, including glioma [26, 27]. Some literatures have revealed several target miRNAs of DANCR in glioma. For instance, DANCR regulated RAB1A expression in glioma by functioning as a ceRNA of miR-634 [27]. In addition, DANCR has been reported to promote glioma malignancy by miR-33a-5p [28]. However, there are no more reports on target miRNAs of DANCR in glioma. Here, we first identified miR-135a-5p as a target of DANCR in glioma cells.

Several researchers have been reported that miR-135a-5p act as a putative tumor suppressor in many cancers by regulating targets expression. For instance, miR-135a-5p inhibited proliferation of neck squamous cell carcinoma via targeting HOXA10 [29]. Moreover, miR-135a-5p regulated cell proliferation, migration and invasion in thyroid carcinoma cells by targeting VCAN 3′UTR [30]. In our study, the results showed that the expression of miR-135a-5p was evidently reduced in glioma tissues and cells. In addition, we explored the relationship between the DANCR and miR-135a-5p in glioma and the results showed that overexpression DANCR conspicuously reduced the level of miR-135a-5p expression, while inhibition of DANCR remarkably elevated the expression of miR-135a-5p in LN229 and U251 cells. These results illustrated that miR-135a-5p was a direct target of DANCR and might negatively correlate with miR-135a-5p. Interestingly, an online software Tarbase v8.0 was carried out, and the results showed that there are potential binding sites of BMI1 in miR-135a-5p, thus we needed to probe their relationship in glioma.

The emerging evidence has suggested that BMI1 acts as an oncogene in different cancers, and its overexpression is associated with poor outcomes in several human cancers. For example, BMI1 promoted invasion and metastasis, and high BMI1 expression was correlated with an advanced stage of breast cancer [31]. In addition, the level of BMI1 mRNA was drastically decreased in the carcinoma tissues, and its elevated expression was involved in human colorectal carcinogenesis via inhibiting the INK4a/ARF proteins [32]. In this study, we revealed that BMI1 expression was markedly upregulated in glioma tissues and cells, which was consistent with previous research [33]. Moreover, we displayed that BMI1 and DANCR were negatively correlated with miR-135a-5p in glioma tissues, and DANCR was positively correlated with BMI1. Thus, these findings indicated that BMI1 might act as an oncogene and played an important role in glioma development. Furthermore, we found that DANCR acted as a ceRNA to regulate BMI1-mediated effects on progression of glioma via sponging miR-135a-5p.

## Conclusion

In conclusion, DANCR was upregulated in glioma tissues and cells. Knockdown DANCR inhibited cell proliferation, migration and invasion in glioma cells. In addition, DANCR could bind to miR-135a-5p, and its elevated expression reversed miR-135a-5p inhibition-mediated progression of glioma. Moreover, DANCR acted as a ceRNA to regulate BMI1 expression and BMI1-mediated effects on progression of glioma by sponging miR-135a-5p. Besides, inhibition of DANCR limited tumor growth by regulating miR-135a-5p and BMI1 expression in vivo. Collectively, DANCR knockdown inhibited cell proliferation, migration and invasion of glioma through regulating miR-135a-5p/BMI1, providing viable therapeutic avenues for treatment of glioma.

## Supplementary information

**Additional file 1: Table S1.** Analysis of the correlation between expression of DANCR and clinicopathological parameters in glioma patients. **Table S2.** Analysis of the correlation between expression of miR-135a-5p and clinicopathological parameters in glioma patients.

## Data Availability

All data generated or analysed during this study are included in this published. Article.
