# Supplementary material for: Long noncoding RNA DANCR knockdown inhibits proliferation, migration and invasion of glioma by regulating miR-135a-5p/BMI1
Source: Cancer Cell Int. 2020 Feb 18;20:53. doi: 10.1186/s12935-020-1123-4 (PMC7029463; doi:10.1186/s12935-020-1123-4)
Supplement: Supplementary file 1 — Additional file 1: Table S1. Analysis of the correlation between expression of DANCR and clinicopathological parameters in glioma patients. Table S2. Analysis of the correlation between expression of miR-135a-5p and clinicopathological parameters in glioma patients. [file 12935_2020_1123_MOESM1_ESM.docx]

Additional file 1: Table S1. Analysis of the correlation between expression of DANCR and clinicopathological parameters in glioma patients

| Variable | Patients, n | DANCR expression | | P-value |
| --- | --- | --- | --- | --- |
|  |  | Low | High |  |
| **Age, years** | 33 | 14 | 19 | 0.325 |
| <60 | 17 | 7 | 10 |  |
| ≥60 | 16 | 7 | 9 |  |
| **Sex** |  |  |  | 0.526 |
| Male | 15 | 6 | 9 |  |
| Female | 18 | 8 | 10 |  |
| **IDH** |  |  |  | 0.389 |
| Wildtype | 30 | 14 | 16 |  |
| Mutant | 3 | 0 | 3 |  |
| **MGMT** |  |  |  | 0.136 |
| Methylated | 5 | 1 | 4 |  |
| Unmethylated | 28 | 13 | 15 |  |
| **Clinical grading** |  |  |  | 0.002 |
| I-II | 14 | 9 | 5 |  |
| III-IV | 19 | 5 | 14 |  |
| **Tumor size** |  |  |  | <0.001 |
| ≥5cm | 17 | 11 | 6 |  |
| <5cm | 16 | 3 | 13 |  |

Additional file 1: Table S2. Analysis of the correlation between expression of miR-135a-5p and clinicopathological parameters in glioma patients

| Variable | Patients, n | miR-135a-5p expression | | P-value |
| --- | --- | --- | --- | --- |
|  |  | Low | High |  |
| **Age, years** | 33 | 18 | 15 | 0.837 |
| <60 | 17 | 8 | 9 |  |
| ≥60 | 16 | 8 | 8 |  |
| **Sex** |  |  |  | 0.628 |
| Male | 15 | 8 | 7 |  |
| Female | 18 | 10 | 8 |  |
| **IDH** |  |  |  | 0.286 |
| Wildtype | 30 | 16 | 14 |  |
| Mutant | 3 | 2 | 1 |  |
| **MGMT** |  |  |  | 0.265 |
| Methylated | 5 | 3 | 2 |  |
| Unmethylated | 28 | 15 | 13 |  |
| **Clinical grading** |  |  |  | <0.001 |
| I-II | 14 | 4 | 10 |  |
| III-IV | 19 | 14 | 5 |  |
| **Tumor size** |  |  |  | <0.001 |
| ≥5cm | 17 | 6 | 11 |  |
| <5cm | 16 | 12 | 4 |  |
